# The effects of a transjugular intrahepatic portosystemic shunt on the diagnosis of hepatocellular cancer

**DOI:** 10.1371/journal.pone.0208233

**Published:** 2018-12-28

**Authors:** Katherine Wong, Katharine Ozeki, Allison Kwong, Bhavik N. Patel, Paul Kwo

**Affiliations:** 1 Division of Gastroenterology and Hepatology, Stanford University School of Medicine, Stanford, CA, United States of America; 2 Department of Radiology, Stanford University School of Medicine, Stanford, CA, United States of America; Texas A&M University, UNITED STATES

## Abstract

**Background and aims:**

Transjugular intrahepatic portosystemic shunt (TIPS) may be placed to treat complications of portal hypertension by creating a conduit between the hepatic and portal vein. The diagnosis of hepatocellular carcinoma (HCC) is typically made by multiphasic imaging studies demonstrating arterial enhancement with washout on arterial, portal venous, and delayed phase imaging. The aim of our study was to determine how the presence of TIPS would affect the imaging diagnosis of HCC.

**Methods:**

This was a single-center electronic database review of all patients who underwent multiphasic imaging with MRI or CT scan for HCC screening between January 2000 and July 2017 and who were subsequently diagnosed with HCC. Data collected included patient demographics, liver disease characteristics including CPT score, MELD-Na, AFP, type of imaging, tumor stage, and lab values at the time of HCC diagnosis. The diagnosis of HCC was made using LI-RADS criteria on contrast-enhanced CT or MR imaging and confirmed by chart abstraction as documented by the treating clinician. Demographic and imaging characteristics for HCC patients with and without TIPS were compared.

**Results:**

A total of 279 patients met eligibility criteria for the study, 37 (13.2%) of whom had TIPS placed prior to diagnosis of HCC. There was no significant difference in demographics or liver disease characteristics between patients with and without TIPS. Compared to cirrhotic patients with no TIPS prior to HCC diagnosis, patients with TIPS had significantly more scans with a longer duration of surveillance until HCC diagnosis. However, LI-RADS criteria and stage of HCC at diagnosis were not significantly different between both groups. There were no differences in outcomes including liver transplant and survival.

**Conclusion:**

The presence of TIPS does not lead to a delayed diagnosis of HCC. It is associated, however, with greater duration of time from first scan to diagnosis of HCC.

## Introduction

Deaths from cirrhosis have increased from 1980 to 2010 [1]. The development of cirrhosis is associated with a higher risk of developing hepatocellular carcinoma (HCC), and an estimated 80–90% of HCC cases occur in the setting of underlying cirrhosis [2]. The worldwide incidence of HCC has increased from 1.4/100,000 person-years in 1976–1980 to 6.2/100,000 person-years in 2011 [3,4,5]. Hepatocellular cancer is one of the few cancers that does not require a tissue diagnosis. Rather, HCC can be diagnosed by multiphasic imaging studies that demonstrate characteristic arterial enhancement with washout on arterial, portal venous, and delayed phase images, which has been standardized using Liver Reporting and Data System (LI-RADS) criteria [6,7]. Since diagnosis of HCC requires characteristic perfusion patterns on imaging, alteration in hepatic blood flow within the liver can potentially lead to a delay in diagnosis of HCC and thus greater disease burden and shortened survival time. The presence of portal vein thrombosis, which alters venous flow within the liver, has been associated with a more advanced stage of HCC at the time of diagnosis [8]. In patients with HCC and portal vein thrombosis, there is a possibility of a lack of characteristic arterial hypervascularity, which may be secondary to compensatory increased arterial supply to the background liver [9]. Furthermore, alterations in the venous supply to the liver could also affect the imaging diagnosis of washout.

Placement of a transjugular intrahepatic portosystemic shunt (TIPS) creates a low-resistance channel between the hepatic vein and the intrahepatic portion of the portal vein, allowing venous blood to travel directly from the portal vein to the hepatic vein while bypassing the liver. TIPS placement treats complications of portal hypertension in cirrhotic patients including refractory ascites and bleeding from esophageal/gastric varices and remains the treatment of choice to decompress the portal system [10]. In the setting of TIPS, where vascular flow in the liver is altered, HCC imaging characteristics have not been defined. The aim of our study was to determine if the presence of a TIPS influences the timing of HCC diagnosis in cirrhotic patients due to the change in blood flow to the liver, which in turn may lead to delayed diagnosis and/or reduced survival.

## Methods

This was a single-center electronic database retrospective review of all patients who underwent dynamic imaging with MRI or CT scan for HCC screening between January 2000 and July 2017 and who were subsequently diagnosed with HCC. The Stanford University Institutional Review Board approved the study. Subjects were identified via the Stanford University electronic data warehouse (STRIDE) by querying ICD-9 (155.0) and ICD-10 (C22.0) codes for hepatocellular carcinoma, and diagnosis of HCC made by clinicians using established imaging-based guidelines and clinical correlation. Data was not anonymized prior to accession but was fully anonymized after data extraction. The review board waived the requirement for informed consent for this study. Patients were excluded from the study if they had a lack of imaging for review or known hepatic mass at referral. Data abstracted included: (1) demographic information (age, gender, race, BMI); (2) presence of other medical comorbidities and risk factors (obesity, diabetes, hypertension, hyperlipidemia, tobacco use, family history of liver cancer); (3) etiology of liver disease; (4) complications related to liver disease including portal vein thrombosis, esophageal variceal bleed, ascites, hepatic encephalopathy, and the indication for TIPS; (5) number, frequency, and type of contrast-enhanced imaging (triphasic CT vs MRI); (6) tumor characteristics on imaging (number of tumors, presence of arterial enhancement, portal venous phase washout, delayed washout, threshold growth, pseudocapsule, vascular invasion, tumor stage); (7) lab values at the time of HCC diagnosis including AFP, serum sodium, creatinine, total bilirubin, and INR; (8) whether patient was listed for liver transplant; and (9) patient survival.

The diagnosis of HCC was documented by the treating clinician, using imaging results reported at the time of diagnosis by a board-certified abdominal radiologist on CT or MRI contrast-enhanced imaging or liver biopsy, and confirmed by chart abstraction where the clinician diagnosed hepatocellular cancer [6]. Surveillance was done every six months in our institution with CT triphasic or MRI with IV contrast. Patient compliance was high. Data abstracted from imaging reports were derived from the 2014 LI-RADs and included lesion size, presence of arterial enhancement (enhancement greater than the background of the liver during the arterial phase), portal venous washout (relative hypodensity or hypointensity of the lesion compared to the background of the liver on CT or MRI, respectively), delayed washout, pseudocapsule (peripheral rim of hyperenhancement in the portal venous or delayed phases), and threshold growth defined as an increase in diameter by a minimum of 5 mm (or ≥50% increase if prior exam ≤6 months, or ≥100% increase if prior exam >6 months, or a new 10mm lesion regardless of time interval) [11]. HCC patients with or without TIPS were compared using chi-square test for categorical variables, t-test for normally distributed continuous variables, and Wilcoxon rank sum test for non-parametric continuous variables. A multivariable quasi-Poisson regression was used to predict time to diagnosis of HCC as the outcome, with age, sex, race, etiology of liver disease, CTP class, number of scans, PVT, and TIPS entered as predictors. Statistical analysis was performed using R. Data reported was median values. A p-value of <0.05 was considered statistically significant.

## Results

**Fig 1:**
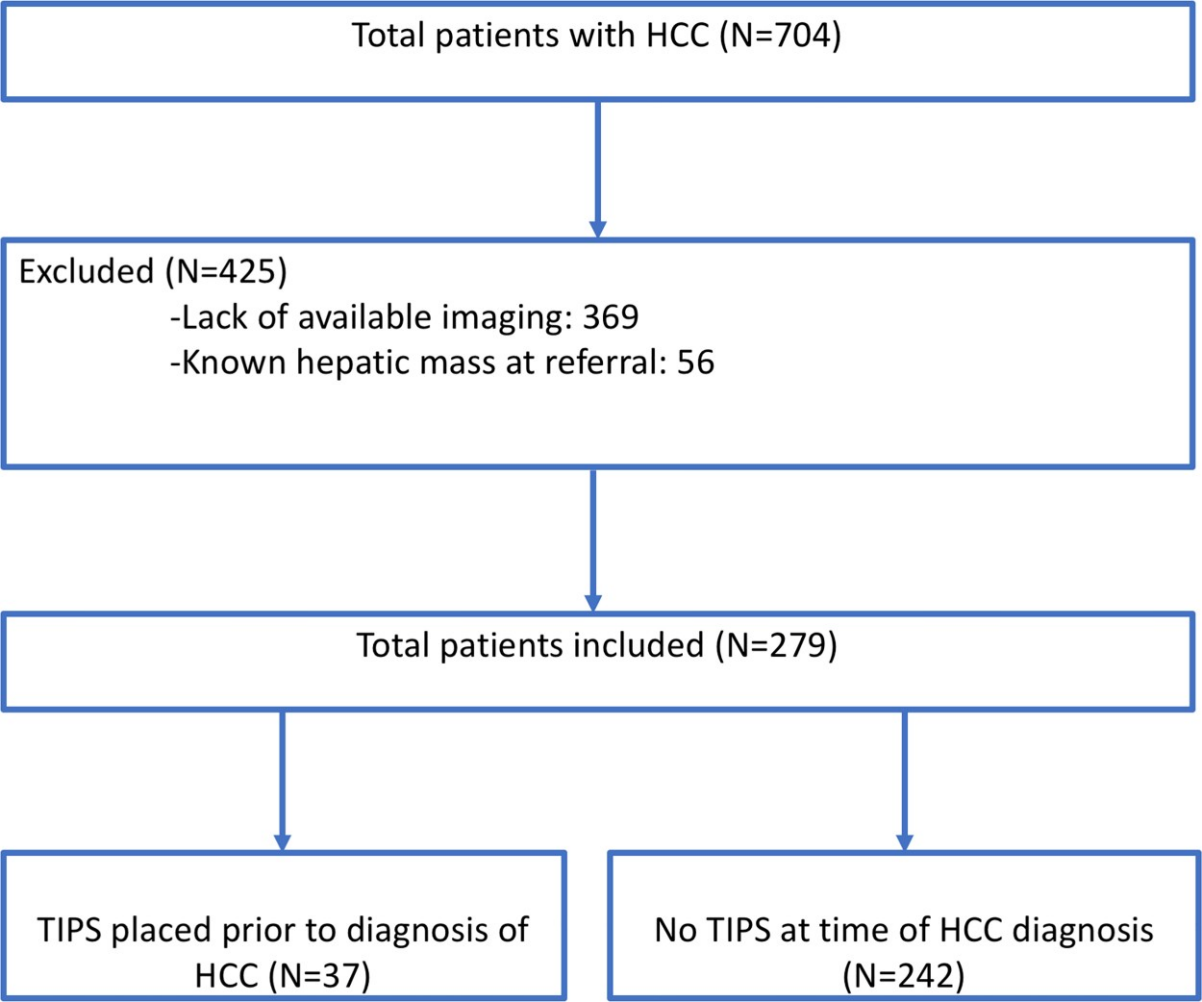
Flowchart of cohort recruitment process with explanations for inclusion or exclusion.

Fig 1 summarizes the cohort recruitment process. A total of 704 patients were identified with a diagnosis of HCC. After exclusion of 369 individuals due to the abovementioned exclusion criteria, 279 subjects met eligibility criteria for the study, 37 (13.3%) of whom had TIPS placed prior to diagnosis of HCC. Table 1 shows demographic data, laboratory values, and liver disease characteristics for all patients, stratified by TIPS status. There was no significant difference between the distribution of liver disease etiology in the TIPS and no TIPS cohorts, the predominant etiology being viral hepatitis C (35.1% vs 46.3%) followed by alcohol (35.1% vs 23.1%). Compared to the patients without TIPS, patients with TIPS were of similar age (62.0 vs 60.8, p = 1.00), similar gender distribution (67.6% vs 70.7% male, p = 0.85) and had similar MELD-Na scores (14 vs 13, p = 0.10) and CTP score (7 vs 6, p = 0.18) at time of HCC diagnosis. A higher mean BMI was noted in patients with TIPS (30.6 ± 6.0 vs 27.6 ± 5.3, p = 0.002) was seen. AFP levels were statistically significantly lower in the TIPS group (5 vs 11, p = 0.002). Compared to the non-TIPS group, patients with TIPS had a higher bilirubin (1.5 v. 1.1 mg/dL, p < 0.01). The group without TIPS also had a lower proportion of patients with hepatic encephalopathy (55% vs 81%, p = 0.005).

**Table 1:** Demographics, laboratory values, risk factors and liver disease characteristics of HCC patients with and without TIPS.

	HCC-TIPS N = 37	HCC-No TIPS N = 242	p-value
Age (IQR)	62.03 [55.26, 65.52]	60.75 [55.09, 66.40]	1.000
BMI (SD)	30.6 (6.0)	27.6 (5.3)	0.002
Male gender (%)	25 (67.6)	171 (70.7)	0.849
Etiology of liver disease (%)			0.050
ETOH	13 (35.1)	56 (23.1)	
HBV	2 (5.4)	37 (15.3)	
HCV	13 (35.1)	112 (46.3)	
NASH	5 (13.5)	11 (4.5)	
Other	4 (10.8)	26 (10.7)	
Race (%)			0.370
African American	0 (0.0)	9 (3.7)	
Asian	6 (16.2)	60 (24.8)	
Other/Unknown	12 (32.4)	72 (29.8)	
White	19 (51.4)	101 (41.7)	
Diabetes (%)	19 (51.4)	110 (45.5)	0.62
Hypertension (%)	22 (59.5)	135 (55.8)	0.81
Hyperlipidemia (%)	12 (32.4)	68 (28.1)	0.73
Smoking (%)	9 (24.3)	75 (31.0)	0.53
Family history of liver cancer (%)	0 (0)	6 (2.5)	0.72
Ascites (%)	30 (81.1)	153 (63.2)	0.052
Variceal Bleed (%)	26 (70.3)	65 (26.9)	<0.001
Hepatic Encephalopathy (%)	30 (81.1)	133 (55.0)	0.005
Creatinine (mg/dL), IQR	1.00 [0.74, 1.20]	0.99 [0.80, 1.19]	0.886
Bilirubin (mg/dL), IQR	1.50 [1.10, 2.20]	1.10 [0.70, 1.90]	0.011
INR, IQR	1.30 [1.20, 1.50]	1.30 [1.10, 1.40]	0.032
MELD, IQR	14.00 [12.00, 16.00]	13.00 [11.00, 16.75]	0.099
AFP (ng/mL), IQR	5.00 [3.00, 8.70]	11.00 [5.00, 64.50]	0.002
CTP score (median [IQR])	7.00 [6.00, 8.00]	6.00 [6.00, 8.00]	0.181

Table 2 compares the imaging characteristics for the two groups. Compared to patients without TIPS, patients with pre-existing TIPS had significantly more scans (3 v. 2, p < 0.001), with a longer duration of surveillance until HCC diagnosis (11 v. 1 month, p < 0.001). However, there was no statistically significant difference in the TIPS cohort for the number of lesions (p = 0.85), LI-RADS 5 score (73% vs 68.6%, p = 0.19), or TNM stage of 3 or greater (13.5% vs 16.1%, p = 0.474). Imaging characteristics were similar in both groups at diagnosis, including arterial enhancement (81.1% vs 90.1%, p 0.18) and delayed venous washout (45.9% vs 54.5%, p = 0.42). Furthermore, there were no differences in liver transplantation (37.8% vs 40.1%, p = 0.94) or survival (73% vs 71.5%, p = 1.00).

**Table 2:** Comparison of imaging findings and staging of HCC in patients with and without TIPS.

	HCC-TIPS N = 37	HCC-No TIPS N = 242	p-value
Number of scans (IQR)	3.00 [2.00, 6.00]	2.00 [1.00, 3.00]	<0.001
Duration of surveillance (months, IQR)	11.00 [1.00, 48.00]	1.00 [0.00, 19.00]	0.001
Number of lesions	1.00 [1.00, 2.00]	1.00 [1.00, 2.00]	0.850
Multiple lesions	17 (45.9)	106 (44.5)	1.000
Arterial enhancements	30 (81.1)	218 (90.1)	0.180
Delayed venous washout	17 (45.9)	132 (54.5)	0.424
Atypical imaging	8 (21.6)	35 (14.5)	0.380
Pseudocapsule	14 (37.8)	66 (27.3)	0.259
MRI Portal venous washout (%)	8 7 (87.5)	33 25 (75.8)	0.807
CT Portal venous washout (%)	29 20 (69.0)	209 118 (51.6)	0.281
Focality (%)			0.489
Missing	0 (0.0)	1 (0.4)	0.662
Infiltrative	0 (0.0)	9 (3.7)	
Multiple	16 (43.2)	99 (40.9)	
Single	21 (56.8)	133 (55.0)	
LI-RADS (%)			0.188
LR-3	4 (10.8)	12 (5.0)	
LR-4	6 (16.2)	64 (26.4)	
LR-5	27 (73.0)	166 (68.6)	
Vascular Invasion (%)			0.335
Missing	2 (5.4)	6 (2.5)	0.372
Macrovascular Invasion	0 (0.0)	11 (4.5)	
Microvascular Invasion	0 (0.0)	3 (1.2)	
None	35 (94.6)	222 (91.7)	
TNM stage at diagnosis			0.387
Missing	0 (0.0)	3 (1.2)	0.474
1	21 (56.8)	121 (50.0)	
2	11 (29.7)	79 (32.6)	
3	0 (0.0)	15 (6.2)	
4	5 (13.5)	24 (9.9)	
Orthotopic liver transplantation	14 (37.8)	97 (40.1)	0.937
Deceased	10 (27.0)	69 (28.5)	1.000

Sensitivity analysis for tumor size greater and less than 2 cm, and with and without PVT, showed similar results—HCC was diagnosed at similar LI-RADS scores and TNM stage regardless of the presence of TIPS (Tables 3 and 4).

**Table 3:** Comparison of imaging findings and staging of HCC larger than 2cm in patients with and without TIPS.

	HCC-TIPS N = 23	HCC-No TIPS N = 153	P-value
Focality (%)			0.410
Infiltrative	0 (0.0)	7 (4.6)	
Multiple	8 (34.8)	64 (41.8)	
Single	15 (65.2)	82 (53.6)	
LI-RADS 2014 (%)			0.647
LR-3	0 (0.0)	5 (3.3)	
LR-4	2 (8.7)	16 (10.5)	
LR-5	22 (91.3)	132 (86.3)	
Vascular invasion (%)			0.443
Missing	0 (0.0)	3 (2.0)	0.550
Macrovascular invasion	0 (0.0)	9 (6.0)	
Microvascular invasion	0 (0.0)	1 (0.7)	
None	23 (100)	140 (93.3)	
TNM Stage (%)			0.165
Missing	0 (0.0)	2 (1.3)	0.244
1	16 (69.6)	73 (47.7)	
2	4 (17.4)	46 (30.1)	
3	0 (0.0)	14 (9.2)	
4	3 (13.0)	18 (11.8)	

**Table 4:** Comparison of imaging findings and staging of HCC less than 2cm in patients with and without TIPS.

	HCC-TIPS N = 13	HCC-No TIPS N = 83	P-value
Focality (%)			0.54
Multiple	7 (53.8)	34 (41.0)	
Single	6 (46.2)	49 (59.0)	
LI-RADS (%)			0.023
LR-3	4 (30.8)	6 (7.2)	
LR-4	4 (30.8)	48 (57.8)	
LR-5	3 (38.5)	29 (34.9)	
Vascular invasion (%)			1.00
Missing	1 (7.7)	2 (2.4)	0.554
Microvascular invasion	0 (0.0)	1 (1.2)	
None	12 (92.3)	80 (96.4)	
TNM Stage (%)			0.120
Missing	0 (0.0)	1 (1.2)	0.247
1	5 (38.5)	48 (57.8)	
2	6 (46.2)	31 (37.3)	
3 or 4	2 (15.4)	3 (3.6)	

In multivariable quasi-Poisson regression, TIPS was not associated with longer time to diagnosis of HCC (p = 0.08). In this model, portal vein thrombosis was an independent risk factor for delayed diagnosis (p = 0.04).

## Discussion

Transjugular intrahepatic portosystemic shunts (TIPS) decompress the portal system to relieve complications of portal hypertension. Multiple meta-analyses and randomized controlled trials have demonstrated the effectiveness of TIPS for secondary prevention of variceal bleeding and treatment of refractory ascites and hepatic hydrothorax. Hepatocellular carcinoma (HCC) is one of the few solid tumor malignancies that does not require tissue diagnosis, but instead relies on characteristic imaging patterns on dynamic imaging. Previous studies have shown that hepatic blood flow is altered by portal vein thrombosis (PVT), resulting in atypical HCC imaging characteristics that did not meet standard diagnostic criteria for HCC, potentially causing significant delays in diagnosis [12,13]. Moreover, no modality has been proven superior with atypical findings of HCC. As a result, hepatocellular cancer patients with PVT were diagnosed at more advanced TNM stage and were less likely to receive liver transplant as curative therapy and was confirmed in this cohort.

A TIPS procedure alters the blood supply to the liver parenchyma by directing blood flow through the stent, which could potentially alter the enhancements and imaging characteristics of the lesion and hepatic parenchyma on the arterial, portal phase, and delayed phase images—all of which are integral to the usual diagnosis of HCC [14–16]. We postulated that such changes in hepatic vasculature could result in delays in the diagnosis of HCC by imaging. The role of TIPS in the management of portal hypertension continues to evolve, and with its recent expanded usage, literature regarding TIPS and its effect on the diagnosis of HCC is limited.

In our study, compared to cirrhotic patients with no TIPS, patients with TIPS had significantly more scans and longer duration of surveillance until diagnosis of HCC, but this did not lead to more advanced presentation or worse clinical outcomes. The greater number of scans in the TIPS group is possibly due to the longer surveillance time. Though not significant, we did note a higher proportion of patients with LI-RADS 3 and 4 in the TIPS group. In addition, there were fewer TNM Stage 1 subjects om the HCC TIPS group though, this did not reach significance. It is possible that this study was underpowered to demonstrate differences between TIPS and non-TIPS HCC patients with lesions <2cm (Tables 5 and 6). Imaging characteristics were similar in both groups at diagnosis, and there was no detectable difference for number of lesions, LI-RADS score, or advanced TNM stage. Furthermore, there were no differences in liver transplantation or survival. Our findings therefore suggest that the presence of TIPS does not lead to a delayed diagnosis of HCC, though there was an increased duration of time from first scan to diagnosis of HCC by imaging.

**Table 5:** Comparison of imaging findings and staging of HCC in patients with PVT, with and without TIPS.

	HCC-TIPS N = 11	HCC-No TIPS N = 85	P-value
Focality (%)			0.343
Infiltrative	0 (0.0)	8 (9.4)	
Multiple	4 (36.4)	40 (47.1)	
Single	7 (63.6)	37 (43.5)	
LI.RADS (%)			0.141
LR-3	0 (0.0)	3 (3.5)	
LR-4	0 (0.0)	20 (23.5)	
LR-5	11 (100.0)	62 (72.9)	
Vascular invasion (%)			0.402
Macrovascular invasion	0 (0.0)	11 (13.3)	
Microvascular invasion	0 (0.0)	1 (1.2)	
None	11 (100.0)	71 (85.5)	
TNM Stage (%)			0.341
1	7 (63.6)	32 (38.6)	
2	3 (27.3)	27 (32.5)	
3	0 (0.0)	12 (14.5)	
4	1 (9.1)	12 (14.5)	

**Table 6:** Comparison of imaging findings and staging of HCC in patients without PVT, with and without TIPS.

	HCC-TIPS N = 27	HCC-No TIPS N = 210	P-value
Focality (%)			0.720
Infiltrative	0 (0.0)	3 (1.4)	
Multiple	12 (44.4)	81 (38.8)	
Single	15 (55.6)	125 (59.8)	
LI-RADS (%)			0.259
LR-3	4 (14.8)	13 (6.2)	
LR-4	6 (22.2)	48 (22.9)	
LR-5	17 (63.0)	149 (71.0)	
Vascular invasion (%)			0.731
Macrovascular invasion	0 (0.0)	3 (1.5)	
Microvascular invasion	0 (0.0)	2 (1.0)	
None	25 (100.0)	199 (97.5)	
TNM Stage (%)			0.532
1	15 (55.6)	112 (53.8)	
2	8 (29.6)	62 (29.8)	
3	0 (0.0)	13 (6.2)	
4	4 (14.8)	21 (10.1)	

Our multivariable regression model did not identify TIPS as an independent risk factor for longer time to HCC diagnosis, but it did confirm findings from prior studies that PVT is associated with delayed diagnosis of HCC (Table 7). The presence of PVT alters hepatic vasculature differently than TIPS placement. Our study also emphasizes the use of LI-RADS as a standardized method to characterize HCC risk of liver lesions in patients with cirrhosis.

**Table 7:** Multivariate analysis for time to diagnosis of HCC as the outcome, with age, sex, race, etiology of liver disease, CTP class, number of scans, PVT, and TIPS entered as predictors.

	Relative Risk	p-value
TIPS at HCC	0.99	0.95
Etiology of liver disease Alcohol HBV HCV NASH Other	1.00 0.74 1.21 0.75 1.40	0.33 0.33 0.36 0.21
Age at diagnosis of HCC	1.00	0.76
Male sex	0.82	0.18
Race White Black Asian Other/Unknown	1.00 0.85 1.61 1.14	0.78 **0.03** 0.42
Log(Number of scans)	4.30	**<0.001**
CTP class A B C	1.00 1.02 1.19	0.90 0.47
Portal vein thrombosis	1.40	**0.02**

Limitations of the study include a relatively small sample size, as this was a retrospective analysis of patient records from a single institution, and all patients referred in for potential liver mass were excluded. Because of the size of this dataset, small differences between groups may have resulted in statistically significant findings (such as INR). In addition, we found paradoxically a lower rate of hepatic encephalopathy in our TIPS cohort, though we did not abstract for use of lactulose or other therapies in this study. However, using this sample, we were able to identify PVT as a risk factor for delayed HCC diagnosis, and did not similarly identify TIPS as an independent predictor—suggesting that the study is adequately powered. The sample is also subject to referral bias, as patients who received TIPS may be have been more closely followed over time. HCC was also diagnosed via imaging and not liver biopsy, which would provide a definitive diagnosis. However, LI-RADS has now superseded liver biopsy in the diagnosis of HCC. We abstracted data from the CT and MR reports using criteria derived LI-RADS 2014 to our entire cohort even though these criteria had not yet been defined during the earlier period of our chart abstraction (2000–2011), though we note no period effect noted. In addition, all scans were prospectively read by radiologists specialized in liver disease at our center and the final diagnosis was confirmed by the clinician evaluating the patient. Analyses involving multiple centers may lead to greater variability and could potentially lead to different results.

Ongoing surveillance for HCC in patients with TIPS remains important. Clinicians may still need to maintain a higher index of suspicion in the presence of TIPS, where hepatic blood flow is altered, and there may be compensatory changes in liver parenchyma and blood supply. The criteria for diagnosis of HCC as defined by the American Association for the Study of Liver Disease (AASLD) or the Organ Procurement and Transplantation Network (OPTN), may be less applicable in cases of HCC in patients with TIPS.

## Abbreviations

TIPS: transjugular portosystemic intrahepatic shunt
PVT: portal vein thrombus
HCC: hepatocellular carcinoma
LI-RADS: Liver Reporting and Data System
AFP: alpha fetoprotein
